# Kappa Index versus CSF Oligoclonal Bands in Predicting Multiple Sclerosis and Infectious/Inflammatory CNS Disorders

**DOI:** 10.3390/diagnostics10100856

**Published:** 2020-10-21

**Authors:** Diana Ferraro, Roberta Bedin, Patrizia Natali, Diego Franciotta, Krzysztof Smolik, Mario Santangelo, Paolo Immovilli, Valentina Camera, Francesca Vitetta, Matteo Gastaldi, Tommaso Trenti, Stefano Meletti, Patrizia Sola

**Affiliations:** 1 Neurology Unit, Azienda Ospedaliero-Universitaria of Modena, 41126 Modena, Italy; valentina.camera@gmail.com (V.C.); vitetta.francesca@aou.mo.it (F.V.); stefano.meletti@unimore.it (S.M.); sola.patrizia@aou.mo.it (P.S.); 2 Department of Biomedical, Metabolic and Neurosciences, University of Modena and Reggio Emilia, 41126 Modena, Italy; bedin.roberta@aou.mo.it (R.B.); 198506@studenti.unimore.it (K.S.); 3 Department of Laboratory Medicine, Azienda Ospedaliero-Universitaria and Azienda Unità Sanitaria Locale, Ospedale Civile, 41126 Modena, Italy; p.natali@ausl.mo.it (P.N.); t.trenti@ausl.mo.it (T.T.); 4 Neuroimmunology Laboratory, IRCCS Mondino Foundation, 27100 Pavia, Italy; diego.franciotta@mondino.it (D.F.); matteo.gastaldi@mondino.it (M.G.); 5 Neurology Unit, Ospedale Ramazzini, Carpi, 41012 Modena, Italy; m.santangelo@ausl.mo.it; 6 Neurology Unit, Ospedale G. da Saliceto, 29121 Piacenza, Italy; p.immovillli@ausl.pc.it

**Keywords:** cerebrospinal fluid, multiple sclerosis, kappa index, oligoclonal bands, free light chains

## Abstract

Background: Cerebrospinal fluid (CSF) kappa free light chains (KFLC) are gaining increasing interest as markers of intrathecal immunoglobulin synthesis. The main aim of this study was to assess the diagnostic accuracy (AUC) of the kappa index (CSF/serum KFLC divided by the CSF/serum albumin ratio) compared to CSF oligoclonal IgG bands (OCB) in predicting Multiple Sclerosis (MS) or a central nervous system infectious/inflammatory disorder (CNSID). Methods: We enrolled patients who underwent a diagnostic spinal tap throughout two years. KFLC levels were determined using a Freelite assay (Binding Site) and the turbidimetric Optilite analyzer. Results: Of 540 included patients, 223 had a CNSID, and 84 had MS. The kappa index was more sensitive (0.89 versus 0.85) and less specific (0.84 versus 0.89), with the same AUC (0.87) as OCB for MS diagnosis (optimal cut-off: 6.2). Adding patients with a single CSF IgG band to the OCB-positive group slightly increased the AUC (0.88). Likewise, the kappa index (cut-off: 3.9) was more sensitive (0.67 versus 0.50) and less specific (0.81 versus 0.97), with the same AUC (0.74) as OCB, for a CNSID diagnosis. Conclusion: The kappa index and CSF OCB have comparable diagnostic accuracies for a MS or CNSID diagnosis and supply the clinician with useful, complementary information.

## 1. Introduction

The detection of cerebrospinal fluid (CSF)-restricted oligoclonal IgG bands (OCB) through isoelectric focusing (IEF) is the current gold standard for determining the presence of intrathecal IgG synthesis. Intrathecal immunoglobulin synthesis occurs following activation of the humoral immune response within the intrathecal compartment and may be present in a number of different infectious or inflammatory central nervous system (CNS) disorders, including Multiple Sclerosis (MS) [1].

A MS diagnosis is possible in the presence of dissemination in space (DIS) and time (DIT) of demyelinating lesions detected with the brain magnetic resonance imaging (MRI), and in the absence of a “better explanation”: Following the latest revisions to the McDonald’s diagnostic criteria [2], the detection of OCB has gained particular importance since it can substitute for the DIT criterion and permits a MS diagnosis in a greater proportion of patients already at onset [3].

Immunoglobulins (Ig) are formed by two heavy chains, which determine their class (IgG, IgM, IgE, IgD and IgA) and by two light chains (either kappa or lambda). Light chains are produced in excess of Ig [4], and the intrathecal synthesis of free light chains (FLC), and in particular of kappa FLC (KFLC) is gaining increasing interest as a possibly more sensitive, less costly and less time-consuming, quantitative marker of intrathecal immunoglobulin synthesis compared to OCB detection. The intrathecal KFLC synthesis can be calculated using different metrics, including the linear kappa index (CSF/serum divided by CSF/serum albumin ratio) and the KFLC intrathecal fraction (KFLC IF), which takes the non-linear relationship of the blood-to-CSF transfer between albumin and KFLC into account [5].

The primary aim of the study was to assess the diagnostic accuracy of the kappa index in comparison with OCB detection in predicting MS or an infectious or inflammatory CNS disease (CNSID) in everyday clinical practice. The secondary outcome was to assess the added value of the KFLC IF calculation compared with OCB and the kappa index.

## 2. Materials and Methods

### 2.1. Patients

In this prospective single-center study, we enrolled all patients who underwent a spinal tap for any diagnostic reason between January 2018 and January 2020 and who had carried out IEF and CSF and serum KFLC measurements as part of the routine diagnostic workup. Information on the patients′ final diagnoses was retrieved from the hospital information system, and patients in whom it was not possible to establish a final diagnosis were excluded from the analysis.

Patients were divided into different groups, based on their final diagnosis: (1) MS, diagnosed according to the 2017 revised McDonald′s diagnostic criteria [2]; (2) Clinically Isolated Syndrome (CIS); (3) other acquired demyelinating CNS diseases (Neuromyelitis Optica Spectrum Disorder—NMOSD, Acquired Demyelinating Encephalomyelitis—ADEM, Radiologically Isolated Syndrome—RIS); (4) CNS inflammatory/paraneoplastic disorders; (5) CNS infectious diseases; (6) epilepsy; (7) Peripheral Nervous System (PNS) diseases; (8) CNS neoplasms; (9) vascular disorders; (10) neurodegenerative disorders; and (11) miscellaneous disorders (including headache, psychiatric diseases, and idiopathic spontaneous CSF hypotension).

The first five disease types constituted the CNSID group; the remaining were grouped in a non-inflammatory disease group (NID).

The study was approved by the Modena Ethics Committee (protocol nr. 1113/2019, date of approval: 14 January 2020).

### 2.2. Laboratory Procedures

At the time of the diagnostic spinal tap, CSF and blood samples were centrifuged at 3000 rpm (rotations per minute) for 10 min and stored in cryogenic tubes at −80 °C within two hours.

CSF and serum samples were analyzed with IEF on an agarose gel followed by IgG immunoblotting.

Serum IgG, CSF IgG, serum albumin, and CSF albumin were analyzed on the Optilite turbidimetric analyzer (The Binding Site Group, Birmingham, UK). The IgG index (CSF/serum IgG quotient—QIgG—divided by the CSF/serum albumin quotient—Qalb), Reiber index ([QIgG − Qlim(IgG)] × serum IgG, where Qlim(IgG) = 0.93 √Qalb^2^ + 6 × 10^−6^ − 1.7 × 10^−3^) and the blood-CSF barrier (B-CSF-B) index damage (Qalb) were then calculated.

CSF and serum kappa FLC levels were determined using the Freelite assay (The Binding Site Group, Birmingham, UK) [6] and using the Optilite turbidimetric analyzer (The Binding Site Group, Birmingham, UK). Assay detection limits were 0.3 mg/L for CSF KFLC.

The kappa index was calculated by dividing the CSF/serum KFLC by the CSF/serum albumin ratio.

In order to minimize false-positive and -negative values at low or high albumin quotients (AQ), respectively, KFLC data was reinterpreted in quotient diagrams (also known as “Reibergrams”, from the name of the developer, Hansotto Reiber) with a hyperbolic reference range using the free software available at: Albaum. Available online: www.albaum.it (accessed on 21 October 2020) for both graphical representations and for the calculation of the KFLC intrathecal fraction (KFLC IF) [(1 − Q lim/Q kappa) × 100 (%)] [5].

### 2.3. Statistical Methods

Comparisons between groups were made using the chi-square and the Wilcoxon’s Mann Whitney test. We calculated sensitivity, specificity, positive predictive value (PPV), negative predictive value (NPV), and diagnostic accuracy of the different biomarkers for a MS diagnosis and for a diagnosis of a CNSID. Diagnostic accuracy was calculated as the area under the Receiver Operating Characteristics (ROC) curve (AUC). With regard to the kappa index, we initially used a cut-off of 5.8, based on a previous study in our laboratory [7], but we also applied the Youden index (sensitivity + specificity − 1) to find optimal cut-off values in the present study population. The test of equality of ROC curve areas was carried out in order to detect significant differences between the examined AUC. Patients with unmeasurable amounts of CSF KFLC were considered as having concentrations equal to 0.15 mg/L (lower detection limit divided by two). Statistical analyses were performed using STATA, version 11 (StataCorp, College Station, TX, USA).

## 3. Results

### 3.1. Patients

**Figure 1:**
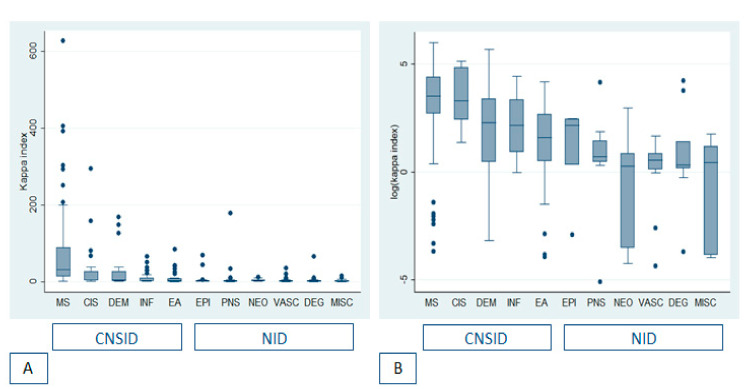
Box-plots showing median kappa index values and interquartile ranges (IQR) amongst different diagnostic subgroups (**A**), and amongst different sub-groups on a logarithmic scale (**B**). Upper whiskers show the largest observation ≤ (third quartile + 1.5 × IQR); lower whiskers show the smallest value ≥ (lower quartile + 1.5 × IQR); black dots show outliers. MS: Multiple Sclerosis; CIS: Clinically Isolated Syndrome, DEM: acquired demyelinating central nervous system (CNS) disorder; INF: CNS infectious disorder; EA: autoimmune/paraneoplastic encephalitis; EPI: epilepsy; PNS: peripheral nervous system disorder; NEO: CNS neoplasm; VASC: vascular disorder; DEG: degenerative neurological disorder; MISC: miscellaneous diagnoses. CNSID: central nervous system inflammatory/infectious disorders; NID: non-inflammatory disorders.

Of 623 patients who had been subjected to a spinal tap for IEF and CSF KFLC measurements during everyday clinical practice in the Neurology Unit of the Azienda Ospedaliero-Universitaria of Modena (Italy) between January 2018 and January 2020, it was possible to formulate a final diagnosis in 540 cases. The final diagnosis was MS in 84 patients (15.6%), CIS in 28 (5.2%), other acquired CNS demyelinating diseases in 23 (4.3%), CNS autoimmune or paraneoplastic diseases in 42 (7.8%), CNS infectious diseases in 50 (9.3%), epilepsy in 28 (5.2%), PNS disorders in 62 (12%), CNS neoplasms in 25 (4.5%), vascular disorders in 61 (11.3%), degenerative neurological disorders in 63 (11.7%), and miscellaneous diseases in the remaining 71 patients (13.1%). Overall, the CNSID group counted 223 (41%) patients and the NID group 317 (59%). Figure 1 shows the distribution of kappa index values amongst different diagnostic subgroups. Table 1 shows demographic and CSF data of the subgroup of patients with MS compared to all other patients.

### 3.2. Concordance between Kappa Index and OCB

Kappa index and CSF OCB were concordant (i.e., presence of at least two CSF-restricted OCB and kappa index ≥5.8 or less than two OCB and kappa index <5.8) in 90% of cases (484/540).

Table 2 shows the diagnoses in non-concordant patients.

### 3.3. Sensitivity and Specificity of OCB and Kappa Index for a MS Diagnosis

Of 84 patients with MS, 71 (85%) patients had CSF OCB, and 75 (89%) had a kappa index ≥5.8.

**Figure 2:**
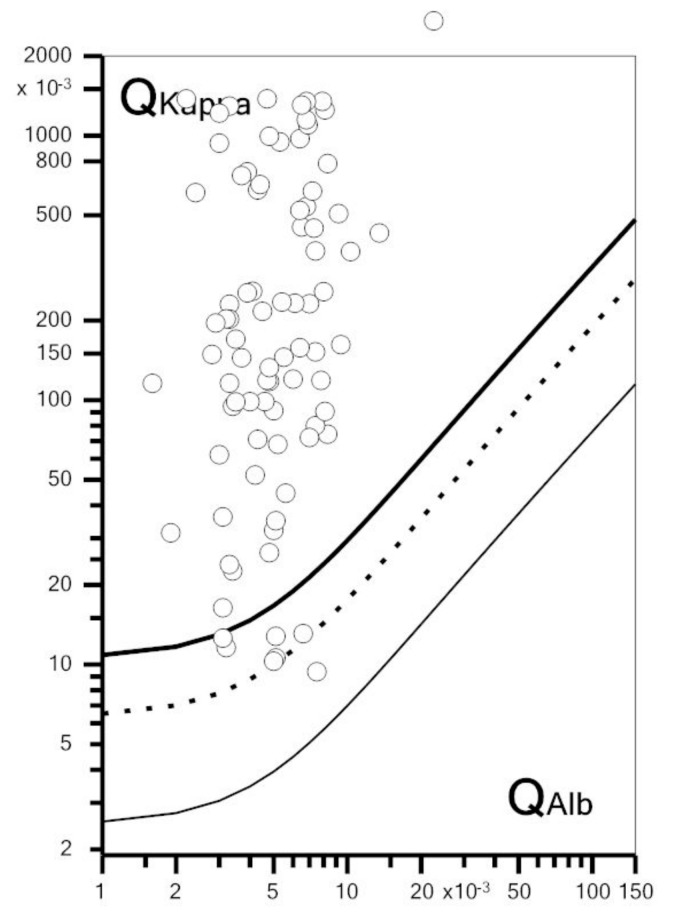
KFLC Reibergram in MS patients. KFLC Reibergram showing MS patients’ CSF/serum KFLC quotients (Q kappa) in relation to their CSF/serum albumin quotients (Qalb). KFLC intrathecal synthesis can be assumed when Q kappa is above the upper line, depicting the hyperbolic border line Q kappa (lim). The dashed line shows the Q kappa mean, and the lower line, the lower limit of the reference range (Q kappa low). The graph was created using the free software available at available at: Albaum. Available online: www.albaum.it (accessed on 21 October 2020).

The optimal kappa index cut-off for a MS diagnosis using the Youden Index was 6.2, but this cut-off did not modify the number of MS patients identified (nr = 75), compared to a cut-off of 5.8. The presence of a KFLC IF >0% identified two additional MS patients (77/84, 92%), while the optimal KFLC IF cut-off value (≥37%), obtained using the Youden Index, identified 76 patients (90%). In non-MS patients, CSF OCB were present in 51 (11%) patients; while a kappa index ≥5.8 or 6.2 was present in 73 (16.0%) and 71 (15.5%) non-MS patients, respectively. Table 3 shows sensitivity, specificity, PPV, NPV and AUC of CSF OCB, of at least one CSF IgG band, of an elevated kappa index value (≥5.8 or ≥6.2), and of an elevated KFCL IF (>0 or >37%) in predicting a MS diagnosis. Briefly, an elevated kappa index and a KFLC IF >37% had a higher sensitivity, lower specificity, and the same AUC compared to OCB for a MS diagnosis. Overall, the highest AUC (0.88) was obtained if patients with a single CSF IgG band were added to the OCB-positive patients. There was no significant difference in the AUC of OCB/KFLC metrics portrayed in Table 3 except for that of a KFLC IF >0%, which was significantly lower compared to the others (*p* < 0.001). Figure 2 shows the KFLC Reibergram in MS patients: all patients except for seven fell in the “intrathecal synthesis” portion of the diagram and had a KFLC IF >0%, thereby lowering the number of false negatives from nine, considering the kappa index, to seven.

### 3.4. Sensitivity and Specificity of OCB and Kappa Index for a CNSID Diagnosis

Of 223 patients with a CNSID, 112 (50%) patients had CSF OCB, and 124 (56%) patients had a kappa index ≥5.8. The optimal kappa index cut-off using the Youden Index was 3.9. A kappa index ≥3.9 was present in 149 (67%) of patients. Again, KFLC metrics turned out to be more sensitive and less specific than OCB, with similar AUC values.

Table 4 shows sensitivity, specificity, PPV, NPV, and AUC of CSF OCB, of at least one CSF IgG band, of an elevated kappa index value (≥5.8 or ≥3.9), and of an elevated KFCL IF (>0 or >11%, according to the Youden Index cut-off) in predicting a CNSID. There was no significant difference in the AUC of the OCB/KFLC metrics portrayed in Table 4.

**Figure 3:**
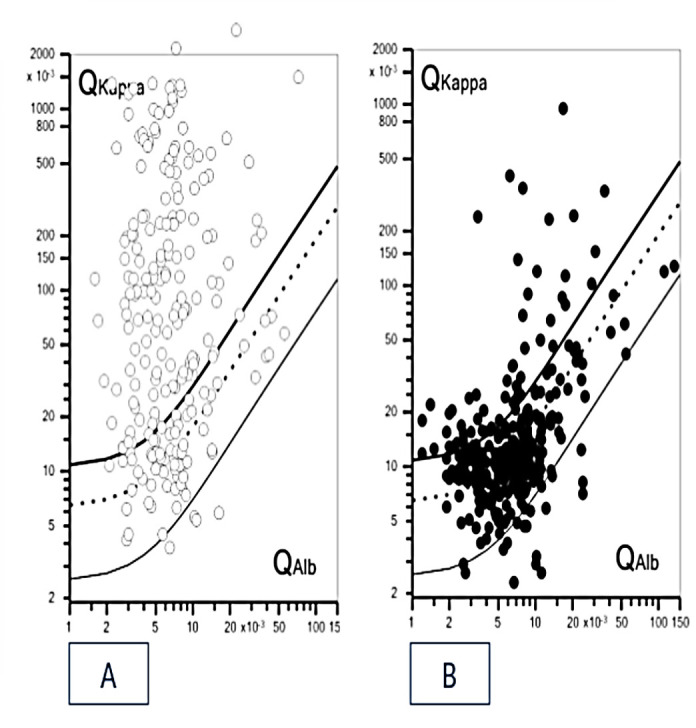
KFLC Reibergrams of CNSID and NID patients. CNSID patients′ data (**A**) and NID patients′ data (**B**) on KFLC Reibergrams showing the CSF/serum KFLC quotients (Q kappa) in relation to the CSF/serum albumin quotients (Qalb). KFLC intrathecal synthesis can be assumed when Q kappa is above the upper line, depicting the hyperbolic border line Q kappa (lim). The dashed line shows the Q kappa mean, and the lower line, the lower limit of the reference range (Q kappa low). Graphs were created using the free software available at Albaum. Available online: www.albaum.it (accessed on 21 October 2020).

Figure 3 shows the KFLC Reibergram of patients with CNSID versus NID. A KFLC IF >0% was present in 154/223 CNSID (69%) patients as opposed to an elevated kappa index, either ≥5.8 or ≥3.9, which was present in 124 (56%) and 149 (67%), respectively.

### 3.5. OCB-Negative Patients and Patients with a Single CSF IgG Band

Thirteen out of 84 MS (16%) patients were OCB-negative; of these, more than one half (7/13; 54%) had a kappa index ≥5.8.

Thirty-one patients had a single CSF IgG band. Approximately half of them (14/31–45%) had a CNSID (MS in seven, CIS in three, acquired CNS demyelinating disorders in two, and one CNS autoimmune and infectious disorder each). Of these, six patients (43%) had a kappa index ≥5.8. Vice-versa, only one patient with a kappa index ≥5.8 did not have a CNSID. Therefore, overall, a positive or negative kappa index correctly classified, as having a CNSID, 22/31 (71%) patients, which is greater than the proportion (14/31; 45%) of correct classification of the single IgG band parameter, amongst patients with a single CSF IgG band.

## 4. Discussion

The main findings of this study are that (a) the kappa index was slightly more sensitive and less specific and has the same diagnostic accuracy as OCB for a diagnosis of MS or of a CNSID, (b) the presence of a KFLC IF >0% increased the sensitivity but had an overall significantly lower diagnostic accuracy for a diagnosis of MS, and a comparable AUC for a diagnosis of a CNSID compared to kappa index/OCB, and (c) the diagnostic accuracy for a MS diagnosis slightly increased, although not significantly so, when adding patients with a single CSF IgG band to the OCB-positive group.

Various studies comparing the diagnostic accuracy of the kappa index and OCB for a MS diagnosis found similar results, with the kappa index being slightly more sensitive and less specific compared to OCB [8,9,10,11], although not all studies are concordant [12,13,14]. The sensitivity and specificity of both kappa index and OCB found in the present study was lower compared to the existing literature, which reports values up to 100% for either sensitivity or specificity [15]. In part, this may be due to the inclusion of a substantial proportion of other CNS inflammatory diseases, which decreases the specificity of both markers in discriminating patients with MS from patients with other diseases.

The best kappa index cut-off value (6.2) for a MS diagnosis was very similar both to the one obtained in a previous study in our laboratory (5.8) [7] and similar to the ones found in two other large studies on this topic (5.9 and 6.6) [8,16]. As expected, for the prediction of a CNSID of any kind, as opposed to only MS, the best kappa index cut-off was lower (3.9), and this should be kept in mind during the differential diagnostic process when assessing a patient with a possible/suspected CNSID. Indeed, the highest kappa index values were found in MS patients (values up to >600), and only patients with MS/CIS or other demyelinating disorders had kappa index values that exceeded the value of 83. Similarly, a study by Cavalla et al. [17] showed that, amongst 371 patients, of which 140 with MS, the highest kappa index values (>100) were observed almost only in MS and were therefore strongly predictive of MS, in patients with the appropriate clinical presentation. Further studies are awaited on the possible association between kappa index values at onset and disease severity and whether they may guide treatment decisions.

It has been suggested to evaluate KFLC with hyperbolic reference range in quotient diagrams as this may reduce the rate of false-positive/-negative cases in the presence of low/high albumin quotients [18] and that the KFLC IF is a more accurate metric compared to OCB and kappa index [11]. In this study, a KFLC IF >0% turned out to be a very sensitive measure, which identified 92% of MS patients, but, due to its low specificity, it had a lower AUC in the prediction of MS, and a similar AUC for the prediction of a CNSID compared to the OCB/kappa index. The optimal cut-off for the KFLC IF (37% for MS and 11% for CNSID) yielded a similar AUC for MS and a marginally, not significantly, higher AUC for the prediction of a CNSID compared to OCB and kappa index (0.76 compared to 0.74).

More than one-half of OCB-negative MS patients had an elevated kappa index, underlining the utility of the kappa index in this patient population, in accordance with previous studies [7,19,20]. The kappa index seems to be useful also in patients with a single IgG band since, in this patient population, an elevated kappa index showed a very high specificity (0.94) and a high PPV (0.86) for the prediction of an infectious/inflammatory CNS disorder, meaning that a positive kappa index in patients with a single IgG band helps to rule in a CNSID.

The addition of patients with a single CSF IgG band to the OCB-positive group increased the likelihood of a MS diagnosis and slightly increased the AUC, underlining the importance of reporting a single CSF IgG band, which was already highlighted in previous studies, since it could be a very early sign of intrathecal synthesis, heralding a future OCB pattern, present, possibly, in patients with a low disease burden [11,21,22].

IEF yields qualitative information, through its different “patterns” [23], on the clonality of intrathecal and systemic IgG, and is a more specific marker of intrathecal synthesis, which may be useful for decreasing the risk of false-positive MS diagnoses. Indeed, the revised McDonald’s MS criteria permit an earlier diagnosis, but at the cost of a lower specificity [24]. Kappa index, on the other hand, supplies quantitative information, which may be useful “per se” (very high kappa index values have been found virtually only in MS), and may capture signs of intrathecal synthesis of other immunoglobulin classes (such as IgM and IgA), which are not detected when searching for IgG OCB and, thanks to its higher sensitivity, it may be useful as a support to the diagnosis of other CNSID, in which intrathecal synthesis may be less “full-blown” than in MS. It may also be particularly useful in the case of single CSF IgG bands, weak bands, and IEF runs that are difficult to interpret. Overall, especially if we consider that the kappa index and OCB are discordant in a proportion of cases, and since they provide complementary information, in our opinion, the most informative approach is to combine both methods in the diagnostic workup of a suspected CNSID.

In fact, in approximately 10% of cases, there was a discordance between CSF-OCB and the kappa index. The discrepant cases were most frequently patients with an elevated kappa index and absent OCB. Interestingly, in the majority of discrepant cases (57%), based on the diagnoses, an intrathecal Ig synthesis was plausible. CNS infections were the most frequent diagnosis in OCB-negative patients with an elevated kappa index, and this may be explained by the fact that KFLC are not specific for the IgG isotype, as they also constitute IgM, IgA, IgD, and IgE. An elevated kappa index could be due, for example, to intrathecal IgM synthesis, which has been shown to be present in a proportion of CIS and MS patients [25,26], but may also represent a primary response in infectious CNS disorders [27,28]. In some infectious diseases, there may be a dominant IgM intrathecal synthesis, such as in neuroborreliosis, in which CSF KFLC seems to be a useful diagnostic biomarker [29,30]. Overall, an isolated intrathecal IgM or IgA synthesis was found in 33 and 14% of cases, respectively, in a large study on 4026 paired serum/CSF samples [31].

A limit of the study is that results were obtained in a single laboratory and that the KFLC assay had a relatively high lower detection limit (0.3 mg/L), which may have reduced the sensitivity of the kappa index. A strength of the study is its relatively large population sample comprising a substantial proportion of other inflammatory CNS disorders, as well as non-inflammatory neurological diseases. The information may, therefore, be useful not only in the context of CIS or suspected MS, but also in real-life everyday neurological practice, in the presence of a suspected CNSID of any kind. Furthermore, different KFLC metrics and thresholds have been evaluated, also in relation to a hyperbolic reference range that takes different albumin quotients into account.

## 5. Conclusions

CSF OCB and kappa index have a comparable diagnostic accuracy in the prediction of MS or of a CNSID. Kappa index has a slightly higher sensitivity and lower specificity than CSF OCB, and both markers supply the clinician with useful, complementary information.

## Figures and Tables

**Table 1:** Demographic and laboratory findings in MS patients versus non-MS patients.

	MS (*n* = 84)	Non-MS (*n* = 456)	*p* Value
Sex (F/M)	54/30	222/236	0.029
Age (years) (mean ± SD)	38 ± 14	57± 20	<0.001
Serum KFLC (mg/L) (mean ± SD)	12.4 ± 5.6	19.2 ± 15.5	<0.001
CSF KFLC (mg/L) (mean ± SD)	4.1 ± 4.5	0.8 ± 2.0	<0.001
Kappa Index (mean ± SD)	78.6 ± 105.8	7.4 ± 22.0	<0.001
KFLC IF (%) (mean ± SD)	78.4 ± 28.6	14.4 ±28.3	<0.001
Patients with KFLC IF > 0, nr (%)	77 (91.7)	132 (29)	<0.001
IgG Index (mean ± SD)	0.8 ± 0.3	0.6 ± 1.1	<0.001
Intrathecal IgG synthesis according to Reiber (mean ± SD)	0.3 ± 1.4	−1.8 ± 3.5	<0.001
CSF/serum albumin (mean ± SD)	5.5 × 10^−3^ ± 2.8 × 10^−3^	9.2 × 10^−3^ ± 11.1 × 10^−3^	<0.001
**IEF pattern**			<0.001
Polyclonal, nr (%)	2 (2.4)	185 (40.5)	
Mirror pattern, nr (%)	4 (4.8)	192 (42.1)	
Single CSF IgG band, nr (%)	6 (7.1)	9 (2.0)	
Mirror pattern + single CSF IgG band, nr (%)	1 (1.2)	15 (3.3)	
CSF-restricted IgG OCB, nr (%)	54 (64.3)	30 (6.6)	
Mirror pattern + OCB, nr (%)	17 (20.2)	21 (4.6)	
Monoclonal gammopathy, nr (%)	0 (0.0)	4 (0.9)	

F: female; M: male, SD: standard deviation, IEF: isoelectric focusing, CSF: cerebral spinal fluid, KFLC: kappa free light chains, OCB: CSF oligoclonal IgG bands, IF: intrathecal fraction.

**Table 2:** Diagnoses in non-concordant patients.

	Kappa Index Positive (≥5.8), OCB-Negative Patients (nr = 41)	OCB-Positive, Kappa Index-Negative (<5.8) Patients (nr = 15)
MS, nr (%)	6 (14.6)	3 (20%)
CIS, nr (%)	1 (2.4)	0
Other acquired CNS demyelinating disease, nr (%)	2 (4.9)	1 (6.6)
CNS autoimmune or paraneoplastic disease, nr (%)	5 (12.2)	3 (20%)
CNS infectious disease, nr (%)	8 (19.5)	3 (20%)
CNS neoplasm, nr (%)	3 (7.3)	1 (6.7)
Neurological degenerative disorder, nr (%)	2 (4.9)	0
Vascular disorder, nr (%)	4 (9.8)	0
PNS disorder, nr (%)	6 (14.6)	2 (13.3)
Epilepsy, nr (%)	0	1 (6.7)
Miscellaneous, nr (%)	4 (9.8)	1 (6.7)

OCB: oligoclonal bands, MS: Multiple Sclerosis, CIS: Clinically Isolated Syndrome, CNS: central nervous system, PNS: peripheral nervous system.

**Table 3:** Diagnostic accuracy of OCB versus KFLC metrics for a MS diagnosis (nr = 84)**.**

	Sensitivity (95%CI)	Specificity (95%CI)	PPV (95%CI)	NPV (95%CI)	AUC (95%CI)
OCB	85 (75–92)	89 (86–92)	58 (49–67)	97 (95–98)	87 (83–91)
OCB + single CSF IgG band	93 (85–97)	84 (80–87)	51 (43–59)	98 (97–99)	88 (85–92)
Kappa Index ≥ 5.8	89 (81–95)	84 (80–87)	51 (42–59)	98 (96–99)	87 (83–90)
Kappa Index ≥ 6.2	89 (81–95)	84 (81–88)	51 (43–60)	98 (96–99)	87 (83–91)
KFLC IF (%) > 0	92 (84–97)	71 (67–75)	37 (30–44)	98 (96–99)	81 (78–85)
KFLC IF (%) > 37	90 (82–96)	83 (80–96)	50 (42–58)	98 (96–99)	87 (83–90)

OCB: oligoclonal bands, CSF: cerebrospinal fluid, PPV: positive predictive value, NPV: negative predictive value, AUC: area under the receiver operating characteristic curve, CI: confidence interval.

**Table 4:** Diagnostic accuracy of OCB versus KFLC metrics for a CNSID diagnosis (nr = 223).

	Sensitivity (95%CI)	Specificity (95%CI)	PPV (95%CI)	NPV (95%CI)	AUC (95%CI)
OCB	50 (44–57)	97 (94–99)	92 (85–96)	73 (69–78)	74 (70–77)
OCB + single CSF IgG band	57 (50–63)	92 (88–94)	82 (75–88)	75 (70–79)	74 (70–78)
Kappa Index ≥ 5.8	56 (49–62)	92 (89–95)	84 (77–89)	75 (70–79)	74 (70–78)
Kappa Index ≥ 3.9	67 (60–73)	81 (77–86)	72 (65–78)	78 (73–82)	74 (70–78)
KFLC IF (%) > 0	69 (63–75)	83 (78–87)	74 (67–80)	79 (74–83)	76 (72–80)
KFLC IF (%) > 11	65 (58–72)	87 (83–91)	78 (74–82)	78 (74–82)	76 (73–80)

OCB: oligoclonal bands, CSF: cerebrospinal fluid, PPV: positive predictive value, NPV: negative predictive value, AUC: area under the receiver operating characteristic curve, CI: confidence interval.
